# Fast and fully automatic calibration of frequency offset for balanced steady-state free precession cardiovascular magnetic resonance at 3.0 Tesla

**DOI:** 10.1186/1532-429X-15-32

**Published:** 2013-04-11

**Authors:** Yu-Wei Tang, Teng-Yi Huang, Wen-Chau Wu

**Affiliations:** 1 Department of Electrical Engineering, National Taiwan University of Science and Technology, Taipei, Taiwan, Republic of China; 2 Graduate Institute of Oncology, National Taiwan University, Taipei, Taiwan, Republic of China

## Abstract

**Background:**

This study proposed a fast and fully automatic calibration system to suppress the dark banding artifacts in balanced steady-state free precession (bSSFP) cardiovascular magnetic resonance (CMR) at 3.0 T.

**Methods:**

Twenty-one healthy volunteers (18 men, 3 women; mean age 24.9 years) participated in this study after providing institutionally approved consent. The optimal frequency was obtained using sweep scans of transition-band low flip-angle bSSFP (bSSFP-L), performed with three conditions: breath-hold plus electrocardiography (ECG) triggering (BH + ECG), breath-hold only (BH), and free breathing (FB). A real-time feedback system was implemented to allow the performing of bSSFP-L calibration scanning and conventional cine bSSFP within one breath-hold. For each scan condition, the optimal phase was estimated using 20-point and 10-point spline fitting.

**Results:**

Linear regression analysis indicated high correlation between the optimal phases obtained using BH and FB and those obtained using BH + ECG (R^2^ = 0.91 to 0.98, n = 21). The optimal phases obtained using 10-point datasets showed high correlation with the 20-point BH + ECG datasets (R^2^ = 0.92 to 0.99, n = 21); although the within-subject coefficient of variation (wsCV) was larger using 10-point fitting. The variation of repeated measurements was largest with FB acquisition and smallest with BH + ECG acquisition. The optimal frequency obtained by offline calculation and by real-time feedback calibration significantly reduced dark-band artifacts in cine bSSFP images (both *p* < .01).

**Conclusions:**

The proposed real-time feedback calibration method for bSSFP imaging is rapid and fully automatic. This method could greatly reduce dark-band artifacts in bSSFP images and facilitate clinical CMR at 3.0 T.

## Background

Cardiovascular magnetic resonance (CMR) has become a practical tool for evaluating cardiac anatomy and function. Of all CMR techniques, balanced steady-state free precession (bSSFP) [1] combines the advantages of sub second scan time, high fluid-tissue contrast, and three-dimensional imaging compatibility. Therefore, the bSSFP sequence has become a major imaging method in clinical CMR protocols [2]. The applications include, but are not limited to, coronary MR angiography [3], accelerated dobutamine stress CMR [4], diagnostics of congenital heart diseases [5], and the detection of myocardial infarction using late gadolinium enhancement CMR [6].

The most prominent artifact uniquely found in bSSFP images is the dark-band artifact arising because of global heterogeneity of the main magnetic field. This occurs because the steady-state signal intensity is a function of the reference offset angle, which is the phase evolution within one repetition time (TR) relative to the radio-frequency (RF) pulse. The location-dependent off-resonance can result in local variations in the SSFP signal and, hence, non-uniformity in signal intensity. Such a phenomenon is referred to as dark-band artifacts. In cardiac imaging for in vivo human study, the magnetic field is generally inhomogeneous at the heart-lung interface [7]. At 3.0 T, the effect is more prominent; dark-band artifacts thus commonly occur in bSSFP-based CMR images.

To solve this problem, localized high-order shimming [8] can reduce the field inhomogeneity. Frequency scout scans [9] can also be used to identify the optimum frequency offset, which further minimizes the presence of the dark-band artifacts in the cardiac structures of interest [9-11]. The frequency scout is a discrete search approach. It acquires bSSFP images with varying RF frequency offsets. The operator observes the image appearances to determine the frequency offset for the subsequent bSSFP scans. However, the precision (i.e., step size of RF frequency offset) of scout scans is limited by the breath-hold time. The user interaction required to identify the optimal frequency offset also delays the clinical routine. If the frequency scout scan is performed only at the beginning of the study, the potential temporal magnetic field instability caused by the heating of the passive shims [12], gradient system [13,14], or chest motion [15,16] might degrade the precision of frequency calibration.

This study presents a method that can rapidly and automatically achieve frequency calibration by proposing a real-time feedback system to obtain the optimal frequency offset immediately after a calibration scan. The study aims to consecutively perform frequency calibration and clinical bSSFP imaging within one breath-hold, and to facilitate clinical routine practice and improve the quality of cardiac images.

## Methods

### Low-flip angle transition-band bSSFP sequence

The transition-band bSSFP with low flip angle (bSSFP-L) detects brain functional activations, as reported by Miller et al. [13]. The sequence removes the RF phase alternation of the conventional bSSFP sequence and uses a relatively small flip angle (typically 3 to 5°). The sequence detects the minute frequency shifts caused by changes in the concentration of deoxyhemoglobin [13,17]. The present study proposes to use the frequency sensitivity of bSSFP-L to detect field inhomogeneity in the cardiac structures of interest. The major steps for estimating the optimal frequency offset using bSSFP-L are performing a sweep scan with varying RF phase angle (β_,_ the phase difference between consecutive RF pulses), plotting an intensity profile of the cardiac region against β, approximating the optimal β corresponding to the global maximum of the intensity profile, and obtaining frequency offset Δ*f* using Δ*f* = β/360*TR*.

### In vivo imaging experiment: bSSFP-L sweep scan and cine cardiac imaging

All imaging experiments were performed using a 3.0 Tesla whole-body MR system (Siemens, Tim Trio, Germany) equipped with a 12-channel cardiac coil. The study was approved by our institution's Internal Review Board Committee. Twenty-one volunteers (18 men, 3 women; aged 24.9 ± 3.17 years) participated in the experiments after providing informed consent. Scout localizers were first obtained and a midlevel short-axis slice was selected. The scan parameters of a bSSFP-L sweep scan were TR/TE = 4/2 ms, flip angle = 4°, matrix = 64 × 64, FOV 280 mm, BW = 555 Hz/pixel, and slice thickness = 6 mm. Each sweep scan yielded 20 images using β = -180 to 162°, with a step size of 18° (equivalently, Δ*f* = - 125 to 112.5 Hz, with a step size of 12.5 Hz), to cover a cycle of potential bSSFP off-resonance phase angles. The steady state was not established before image acquisition. Shimming was accomplished before the patient was first scanned with the bSSFP-L sequence. The operator positioned the FOV for the heart region to be located at the center of the acquired image. Three types of bSSFP-L sweep scan were performed: breath-hold plus ECG triggering (BH + ECG, trigger delay = 130 ms), breath-hold (BH), and free breathing (FB). The BH + ECG method was considered the reference standard to justify if BH or FB methods are appropriate for frequency calibration using the bSSFP-L method. When performing the breath-hold experiment, the operator asked participants to hold their breaths at the end of expiration. The total acquisition times for BH + ECG, BH, and FB were 20 heartbeats, 5.4 s, and 5.4 s, respectively. Each type of sweep scans was performed 3 times to assess reproducibility. All sweep scans were performed consecutively to prevent frequency drift over time. After the sweep scans, the optimal Δ*f* was calculated using an external computer. The calculation procedures are presented in a later section.

### Image processing: automatic region of interest selection and calculating optimal frequency offset

**Figure 1:**
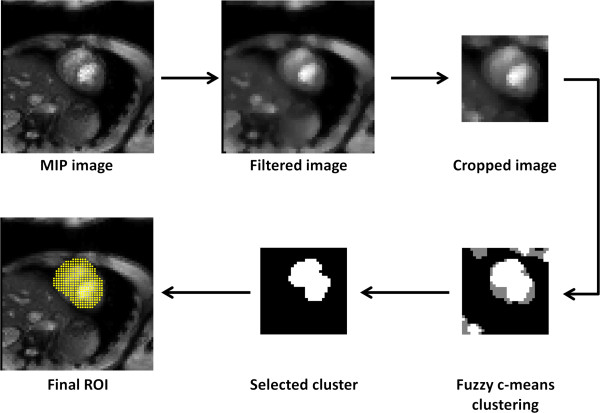
**Procedures of automatic ROI selection.** A MIP image is first generated from all the acquired sweep scan images. After applying a median filter, the procedure selects a square region consisted of 25 × 25 pixels and uses FCM clustering to distribute pixels into 3 groups according to the signal intensity. The group with the highest averaged intensity generates a binary mask. The binary mask then underwent erosion and subsequent dilation to remove small objects and connect neighboring objects in the mask. Finally, the procedure selected an object with the center of mass closest to the center of FOV as heart ROI (indicated as yellow dots).

In total, 9 datasets for each participant were transferred offline to a personal computer (PC) and processed using Matlab® (Mathworks, Natick, MA, USA). An automatic algorithm was then implemented to define a region of interest (ROI) encompassing most of the heart region. Figure 1 displays the procedures of automatic ROI selection. First, the procedure calculated a maximum intensity projection (MIP) image of a sweep scan dataset. The MIP procedure was performed on images acquired using different Δ*f* values. The FOV of the image was positioned for the heart to be located at the center of the image. The subsequent procedures processed only the pixels inside a square area with 25 × 25 pixels at the image center. Second, fuzzy C-means (FCM) [18] was used to segment pixels in the MIP image into 3 clusters according to their pixel intensities. The cluster with highest pixel intensities was selected to produce a binary mask. The mask underwent erosion and dilation to remove small objects and connect neighboring objects. Finally, the procedure selected an object with its center of mass closest to the center of the FOV as the heart ROI.

Subsequently, the procedure calculated the averaged intensities over the heart ROI of a bSSFP-L image dataset to plot a signal intensity curve against Δ*f* (hereafter termed sweep scan curve). The samples of the sweep scan curve were increased using the cubic spline interpolation technique. Finally, Δ*f*, corresponding to the peak of the interpolated curve, was regarded as the optimal frequency offset. In real-time calibration, a shorter total acquisition time facilitates its clinical application. The procedures were, therefore, repeated to calculate Δ*f* using only 10 images (Δ*f*  = - 125 to 100 Hz, with a step size of 25 Hz) of the sweep scan. The 10-image datasets are referred to as 10-point (10-pt) datasets hereafter.

### Statistical analysis

Within-subject coefficient of variation (wsCV) was calculated to assess the reproducibility of Δ*f* estimation for 6 combinations of image acquisition methods (BH + ECG, BH, and FB) and curve-fitting schemes (20-point and 10-point spline fitting):

$$\mathrm{wsCV}=\sqrt{\frac{1}{N}\sum_{i=1}^{N}{\left(\frac{\sigma_{i}}{{\bar{X}}_{i}}\right)}^{2}}$$

where σ_i_ and ${\bar{X}}_{i}$ were the standard deviation and mean, respectively, of the three repeats on subject i. N was the number of subjects included in the calculation. Out of 126 sets of repeated measurements (21 subjects × 6 = 126), four were excluded because their means were very close to zero where wsCV is mathematically undefined. The combination of BH + ECG acquisition and 20-pt spline fitting was considered as the reference standard to which other five methods were compared using linear regression analysis including the four cases previously removed from wsCV calculation.

### Real-time feedback system: automatic frequency calibration and bSSFP imaging

**Figure 2:**
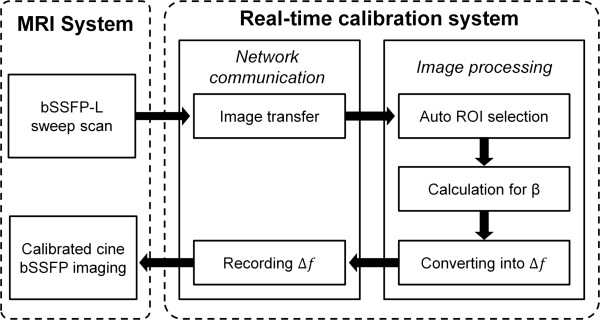
**The framework of the proposed real-time feedback calibration system.** The reconstructed bSSFP-L images are transferred to an external PC synchronously through an Ethernet network and the PC processes the sweep scan datasets, obtains the optimal β, converts β into frequency offset and records it in an F-file. Finally, the subsequent bSSFP imaging program reads F-file and adjusts the offset frequency of the imaging sequence to achieve real-time calibration without user interactions.

The real-time feedback system consists of 3 major parts: image acquisition, network communication, and image processing (Figure 2). The calibration algorithm in this study was performed on a PC with an Intel Core i7 processor. The MR system and PC communicated through an Ethernet connection and a network file system. The image reconstruction system of the MR scanner was programmed to transfer the images to the PC immediately after image reconstruction. After image acquisition of the sweep scan, the PC-based MATLAB program automatically calculated the optimal frequency, and then recorded it in a file named F-file. The subsequent bSSFP imaging sequence was modified to perform imaging using the frequency stored in the F-file.

### Real-time feedback system: in vivo experiments

Ten participants (male, aged 24.8 ± 4.9 years) participated in the real-time calibration experiments. They underwent three multiphase cine bSSFP scans without frequency calibration, with frequency-scout calibration, and with bSSFP-L calibration. The cine scans acquired the same short-axis slice using the following scan parameters: TR/TE = 3.6/1.8 ms, flip angle = 49°, matrix = 256 × 256, FOV = 300 mm, bandwidth = 930 Hz/pixel, ECG-gated, number of cardiac phase = 25, and two-fold GRAPPA [19] acceleration with 44 reference lines. For the bSSFP-L real-time feedback calibration, a 10-pt bSSFP-L sweep scan (Δ*f* = -125 to 100 Hz, with a step size of 25 Hz, scan time = 2.7 s) and a cine bSSFP scan were performed consecutively with a 1-s time delay. The two imaging sequences were set to acquire the slice at an identical position. A midlevel short-axis slice was selected for each volunteer. Additional slices (short axis, long axis, four-chamber view, right ventricular outflow tract) were acquired for two of the volunteers. The participants were asked to hold their breath during both scans. To calculate the total processing time of the feedback system, a trigger signal synchronized with the last excitation pulse of the bSSFP-L sequence was transmitted using fiber-optic cabling to the dedicated PC.

### Approximate assessment of dark-band artifacts

To assess the dark-band artifacts of the three cine datasets (without frequency calibration, with frequency-scout calibration, and with bSSFP-L calibration), an ROI covering the myocardium and blood pool of the left ventricle (LV) was manually outlined for each cardiac phase of the cine datasets. The average ROI signal intensity was also calculated. A pixel inside the ROI was classified as part of a dark-band artifact when the signal intensity was below a certain threshold. Although the simple bisection method can be employed to detect the banding artifacts, it may not be able to distinguish dark-band pixels from tissues with inherently low signals such as the papillary muscle. Therefore, we used six thresholds to assess the dark-band pixels to compare the performance of the frequency calibration methods. Thresholds #1, #2, and #3 were 10%, 15%, and 20% of the average signal intensity of the ROI in each dataset, respectively. Threshold #4 was the average value of the threshold #1 values for the three datasets (without frequency calibration, with frequency-scout calibration, and with bSSFP-L calibration). Similarly, thresholds #5 and #6 were obtained from thresholds #2 and #3, respectively.

## Results

**Figure 3:**
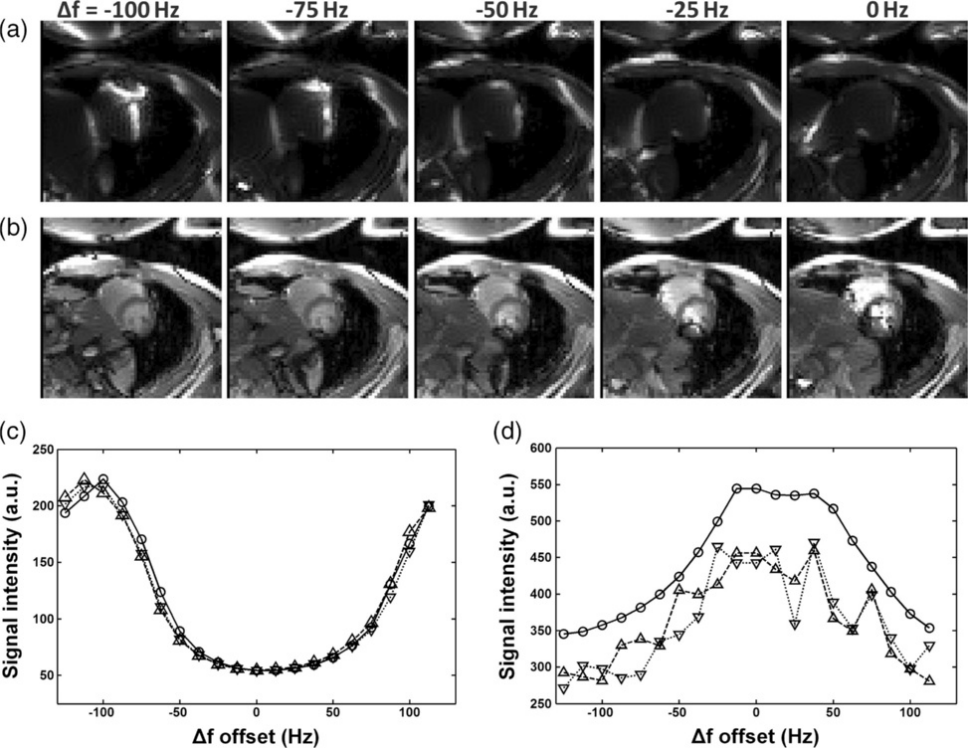
**The selected images obtained using sweep scan with (a) bSSFP-L (b) bSSFP. **Subfigures (**c**) and (**d**) show the ROI-averaged intensity curves acquired using the bSSFP-L sweep scan and the conventional bSSFP frequency scout (BH + ECG: circle, BH: triangle, FB: inverted triangle), respectively.

Figure 3(a,b) displays typical sweep-scan images obtained (BH + ECG, Δ*f* = - 100 Hz to 0 Hz) using bSSFP-L and bSSFP. As shown in the bSSFP-L images (Figure 3a), thin bands with significantly high intensities shift against Δ*f*. In the bSSFP images (Figure 3b), dark bands shift against Δ*f*. When Δ*f* = - 100 Hz, the bright band of the bSSFP-L image is located at the center of LV and the corresponding bSSFP image appears nearly free of dark-band artifact around the LV region. Figure 3(c) and 3(d) show the ROI-averaged intensity curves of 20-pt sweep-scan images obtained under the 3 conditions (BH + ECG, BH, and FB). The curves acquired using the bSSFP-L sweep scans under different conditions appear similar. In contrast, the sweep scan curves obtained using the conventional bSSFP frequency scout without an ECG trigger or breath-hold shows prominent oscillations. In addition, the peaks of the bSSFP-L curves (Figure 3c) are located around -100 Hz, which is approximately the optimal frequency of bSSFP imaging. The results suggest that the bSSFP-L method is more suitable for automatic frequency calibration then the bSSFP method.

**Figure 4:**
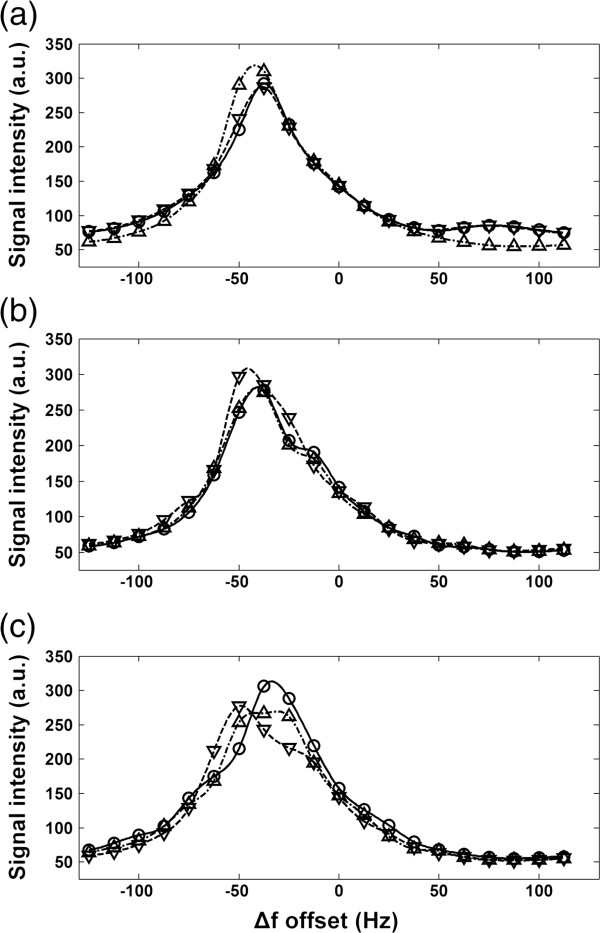
**Typical 20-pt sweep scan curves obtained using bSSFP-L under three conditions (a) BH + ECG, (b) BH, (c) FB.** For each condition, three repeated sweep scans were performed. From (**a**) to (**c**), the reproducibility appear progressively lower.

Figure 4 displays typical ROI-averaged intensity curves of 20-pt sweep-scan images obtained using bSSFP-L. The BH + ECG curves of the three repeated measurements appear almost identical and the calculated optimal Δ*f* value was -39.4 ± 2.6 Hz. This observation supports that the sweep scan with BH + ECG is a reproducible method with which to obtain the optimal Δ*f*. The BH + ECG datasets were regarded as reference standards to assess the reproducibility and accuracy of the other datasets. In the BH dataset (without ECG), the curves exhibit subtle variations, obtaining a similar Δ*f* of -40.5 ± 3.0 Hz compared with BH + ECG datasets. In the FB dataset, the curves show relatively large variation and the obtained Δ*f* was -44.7 ± 10.1 Hz.

**Figure 5:**
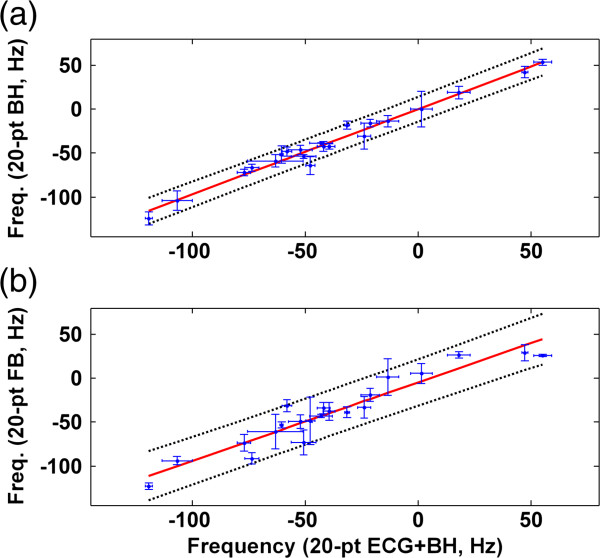
**Group analysis of 20-pt bSSFP-L sweep scan datasets (a) 20-pt BH vs. 20-pt BH + ECG, (b) 20-pt FB vs. 20-pt BH + ECG(blue dot and error bar: mean and standard deviation of the** Δ***f *values obtained in three repeated scans, red line: linearly fitted lines, black dotted line: 95% prediction interval).**

**Figure 6:**
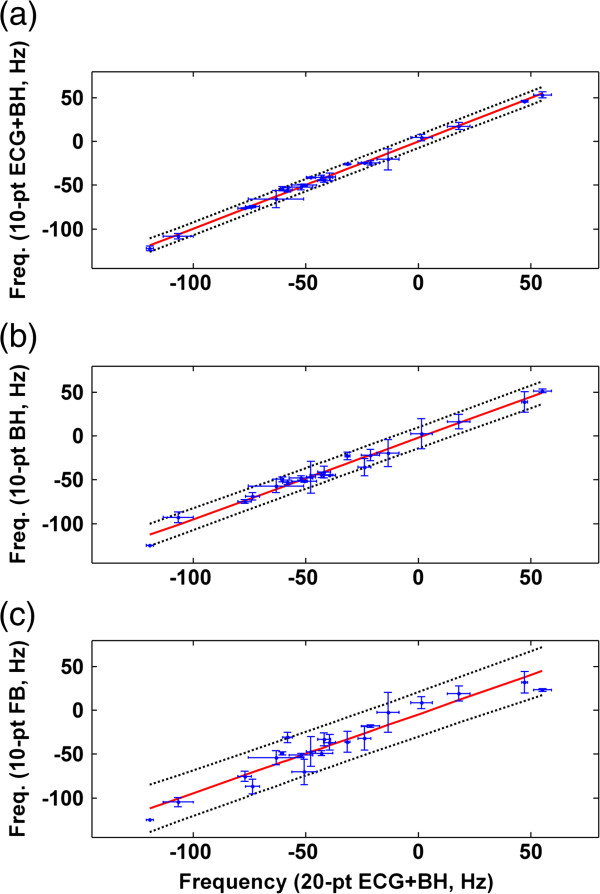
**Group analysis of 10-pt sweep scan datasets against 20-pt BH + ECG datasets (a) 10-pt BH + ECG vs. 20-pt BH + ECG, (b) 10-pt BH vs. 20-pt BH + ECG, (c) 10-pt FB vs. 20-pt BH + ECG (blue dot and error bar: mean and standard deviation of the** Δ***f *values obtained in three repeated scans, red line: linearly fitted lines, black dotted line: 95% prediction interval).**

Figure 5 compares the optimal Δ*f* values obtained using 20-pt sweep scans under different conditions. Linear regression analysis of comparison for BH versus BH + ECG indicates high correlation (R^2^ = 0.98); for FB versus BH + ECG, it also indicates high correlation (R^2^ = 0.91). Figure 6 compares the optimal Δ*f* values obtained using the 20-pt sweep scan with BH + ECG and three 10-pt data sets (BH + ECG, BH, FB). Linear regression analysis revealed that the optimal Δ*f* values obtained by the 10-pt datasets are in agreement with the optimal Δ*f* values obtained by the 20-pt sweep scan with BH + ECG (R^2^ = 0.91 to 0.99). Table 1 lists the parameters obtained in the regression analyses.

**Table 1:** Linear regression analysis against 20-pt BH + ECG datasets

**Dataset**	**Slope**	**R**^**2**^	**95% C.I. (Hz)**
20-pt			
BH	0.97	0.98	6.93-7.65
FB	0.89	0.91	13.02-14.38
10-pt			
BH + ECG	1.00	0.99	3.58-3.93
BH	0.93	0.98	4.89-6.50
FB	0.90	0.92	12.46-13.75

**Figure 7:**
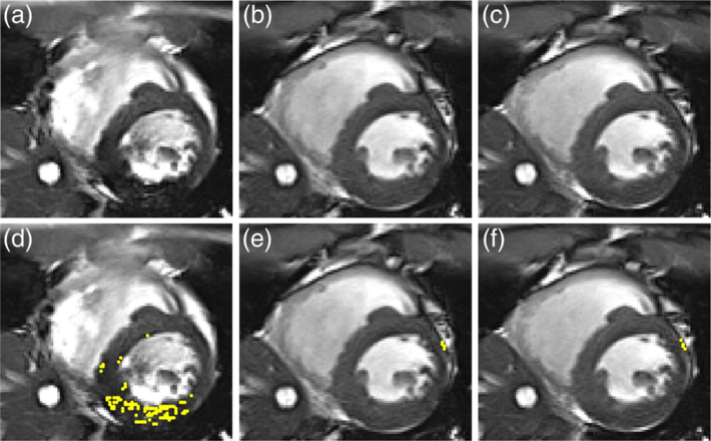
**Locally enlarged images of a man aged 22 years obtained using cine bSSFP imaging: (a) without frequency calibration, (b) with frequency-scout calibration, and (c) with real-time 10-pt bSSFP-L BH calibration (324 ms after the ECG R-wave).** The yellow pixels in (**d**-**f**) are considered dark-band artifacts within the manually selected LV ROI in (**a**-**c**). This comparison shows that both calibration methods, that is, frequency scout and real-time bSSFP-L, substantially reduced the dark-band artifacts.

Figure 7 shows selected cine bSSFP images obtained from a volunteer with and without frequency calibration. The regions marked by yellow dots are approximately estimated as dark-band artifacts. The operator outlined a ROI covering the blood pool and walls of the LV for each cardiac phase of the cine bSSFP images. The results of a group comparison (n = 10) using dark-band artifacts estimated with six thresholds showed that the frequency-scout method and the 10-pt BH bSSFP-L method significantly reduced the dark-band artifact (*p* < .01, paired Student’s *t* test). The performances of the two frequency calibration methods were similar (*p* > .7, paired Student’s *t* test). A high correlation was observed between the offset frequencies obtained using the two calibration methods (R^2^ = 0.98, slope = 0.95).

**Figure 8:**
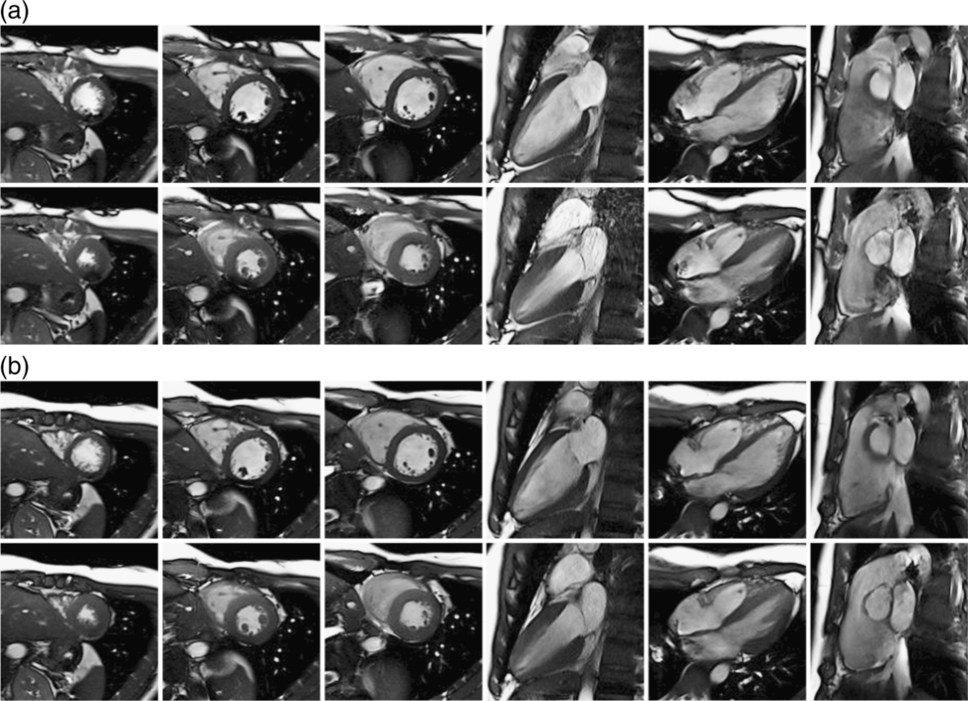
**Selected phases (diastole: upper row, systole: lower row) of cine bSSFP images (left to right: apical short axis, midlevel short axis, basal short axis, left two-chamber view, four-chamber view, right ventricular outflow tract) obtained without (a) and with the 10-pt bSSFP-L BH frequency calibration (b).** The calculated frequency offsets for six slice positions are (left to right:-75 Hz, -42 Hz, -51 Hz, -46 Hz, -49 Hz,-51 Hz). Performing cine bSSFP imaging with automatically calculated frequency offset significantly reduced dark band artifacts in the cardiac structures of interest.

Figure 8 displays examples of real-time feedback-calibrated bSSFP imaging. It shows selected images acquired with different slice orientations using a conventional cine bSSFP imaging sequence without and with frequency calibration (Figure 8a and 8b). The processing time needed for image reconstruction on the scanner and network image transmissions was approximately 300 ms (10 trials). The processing time, including calculating the Δ*f* optimization algorithm on the PC and the network feedback, was approximately less than 100 ms (10 trials). For real-time feedback-calibrated bSSFP imaging, group analysis (n = 7) indicated significant reduction in artifact (*p* < .01, paired Student’s *t* test).

Table 2 presents the results of the reproducibility tests. The wsCV is the lowest for the reference standard (BH + ECG, 20-point spline fitting), and increased with the removal of motion control (ECG gating and breath-hold) and the use of fewer data points for curve fitting.

**Table 2:** Summary of within-subject coefficient of variation (wsCV) computed for six combinations of imaging acquisition methods (BH + ECG, BH, FB) and curve-fitting schemes (20-pt and 10-pt spline fitting)

**(%)**	**BH + ECG**	**BH**	**FB**
20-pt	13.4	21.1	23.7
10-pt	23.3	26.2	30.9

## Discussion

This study implemented a fully automatic and rapid frequency calibration method to reduce dark-band artifacts in bSSFP imaging, by taking advantage of the sensitivity the bSSFP-L sequence to minute frequency drifts. Results indicated the feasibility of performing the sweep scan obtaining Δ*f* without ECG triggering. The entire calibration time was further reduced by using a sparser sampling scheme (10-pt sweep scan) and a real-time feedback system. The shortened calibration time allows this method to serve as a pre-adjustment module for bSSFP imaging sequences. It is feasible to perform the calibration scan and bSSFP imaging within one breath-hold.

Considering the breath-hold period, this study used 20-pt sweep scans with BH + ECG to increase the precision of Δ*f* estimation, using the acquired 20-pt BH + ECG datasets as reference standards with which to judge the performance of the remaining data sets. Quantitative assessment of cine data demonstrated a significant reduction in dark-band artifacts following calibration using the 20-pt BH + ECG method. The linear regression analysis showed high correlation between the 20-pt BH datasets and the 20-pt BH + ECG datasets (R^2^ = 0.98, slope = 0.99). Variation analysis of repeated measurements supports that the reproducibility of the 20-pt BH method is comparable to that of the 20-pt BH + ECG method. This result indicates that cardiac motion minimally affects the signal intensities of bSSFP-L images. Using ROI-based analysis, the optimal Δ*f* can be accurately obtained without ECG triggering. Without using ECG triggering, the image acquisition time of the 20-pt sweep scan reduces from 20 heartbeats to 5.4 s. The 20-pt FB datasets show a high correlation with the 20-pt BH + ECG datasets, albeit with lower slope (R^2^ = 0.91, slope = 0.89) and relatively higher variation of the repeated measurements (wsCV = 23.7%). This result indicates that respiratory motion reduces precision and reproducibility of the Δ*f* measurement. These comparisons suggest that the BH method, which provides greater precision (R^2^ = 0.98, slope = 0.97, wsCV = 21.1%) and fast data acquisition, is the optimal choice for frequency calibration using bSSFP-L sweep scans. The FB method could be particularly useful in patients with limited breath-hold capacity.

To further reduce image acquisition time, this study investigated the feasibility of obtaining accurate frequency offset with a sparser sampling scheme, the 10-pt sweep scan method. Analyses of linear regression and reproducibility suggested that the 10-pt BH method is capable of producing accurate and reliable Δ*f*, which is comparable to that obtained using the 20-pt BH + ECG method. The shortened acquisition time of the 10-pt BH method allows the performing of real-time frequency calibration and clinical bSSFP imaging within one breath-hold. Using the 10-pt BH method shortened the total data acquisition time of the Δ*f* measurement to 2.7 s. The total processing time of the feedback system (data networking and software calculation of Δ*f*) is less than 1 s. The results indicate that the real-time feedback system can calibrate frequency offset for the subsequent conventional bSSFP imaging and substantially reduce the dark-band artifact in the bSSFP images. Frequency calibration for each bSSFP scan in the clinical protocol is, therefore, feasible. It is possible to obtain optimal Δ*f* values for different slice locations and orientations. In addition, calibration of each bSSFP scan in the clinical protocol could potentially enable the tracking of frequency drift during a long study.

Using the 10-pt method, the frequency resolution is 25 Hz (step size of β = 36° and TR = 4 ms). In clinical practice, the frequency scout method obtains optimal results while matching the parameters of the scout scan and bSSFP imaging, such as TR and bandwidth. The purpose of the proposed bSSFP-L method is to calculate the off-resonance frequency around the heart. Theoretically, this frequency would be unaffected by the modulation of imaging parameters. However, for other factors that would affect the bSSFP signal such as the eddy current, RF imperfection, and flow effect, matching the imaging parameters of the cine sequence should be an optimal approach.

Our data indicate that the performance of the proposed bSSFP-L method matches that of the conventional frequency scout method. The main advantages of the bSSFP-L method are the fully automatic procedure and rapid acquisition. The sweep scan curve obtained using the bSSFP-L sequence without an ECG trigger is affected minimally by cardiac motion. In contrast, the sweep scan curve obtained using the conventional bSSFP frequency scout without an ECG trigger shows prominent oscillations. Consequently, it is relatively difficult to achieve an automatic procedure for the bSSFP datasets, especially when a sparse sampling scheme (10-pt method) is used and the ECG trigger is turned off.

The limitation of this study is that the validation of the proposed method is performed only on healthy young adults. A potential pitfall of this method is the automatic ROI selection procedure. Whether the ROI selection algorithm is still robust on patients with cardiac dysfunction remains unanswered. The proposed algorithm requires the operator to position the heart region at the center of the FOV. This could lead to wraparound artifacts, especially when parallel imaging is used. Nonetheless, it is potentially feasible to implement a tool similar to the FOV of the “localized volume shim” on the user interface of the MR scanner console to position a rectangle covering the heart region. This procedure could further improve the robustness of the frequency calibration and avoid the restriction of the FOV position.

## Conclusion

In summary, the proposed real-time calibration method, which rapidly and accurately optimizes the frequency offsets of bSSFP imaging, is fully automatic and can facilitate clinical bSSFP cardiac imaging and improve image quality, especially at 3.0 T.

## Competing interests

The authors and the institutions have no conflicts of interest related to this paper.

## Authors’ contributions

TYW, HTY and WWC have made substantial contributions to conception and design, or acquisition of data, or analysis and interpretation of data; TYW, HTY and WWC have been involved in drafting the manuscript or revising it critically for important intellectual content; TYW, HTY and WWC have given final approval of the version to be published. TYW and HTY carried out the bSSFP sequence design and feedback system. HTY, WWC developed the automatic image processing algorithm and performed the statistics. All authors read and approved the final manuscript.
